# Effects of Edaravone, a Free Radical Scavenger, on Photochemically Induced Cerebral Infarction in a Rat Hemiplegic Model

**DOI:** 10.1155/2013/175280

**Published:** 2013-06-17

**Authors:** Satoshi Ikeda, Katsuhiro Harada, Akihiko Ohwatashi, Yurie Kamikawa

**Affiliations:** ^1^Department of Rehabilitation and Physical Medicine, Graduate School of Medical and Dental Sciences, Kagoshima University, 8-35-1 Sakuragaoka, Kagoshima 890-8506, Japan; ^2^School of Medical Sciences, Faculty of Medicine, Kagoshima University, 8-35-1 Sakuragaoka, Kagoshima 890-8544, Japan

## Abstract

Edaravone is a free radical scavenger that protects the adjacent cortex during cerebral infarction. We created a hemiparetic model of cerebral thrombosis from a photochemically induced infarction with the photosensitive dye, rose bengal, in rats. We examined the effects of edaravone on recovery in the model. A total of 36 adult Wistar rats were used. The right sensorimotor area was irradiated with green light with a wavelength of 533 nm (10 mm diameter), and the rose bengal was injected intravenously to create an infarction. The edaravone group was injected intraperitoneally with edaravone (3 mg/kg), and the control group was injected with saline. The recovery process of the hemiplegia was evaluated with the 7-step scale of Fenny. The infarcted areas were measured after fixation. The recovery of the paralysis in the edaravone-treated group was significantly earlier than that in the untreated group. Seven days later, both groups were mostly recovered and had scores of 7, and the infarction region was significantly smaller in the edaravone-treated group. Edaravone reduced the infarction area and promoted the functional recovery of hemiparesis from cerebral thrombosis in a rat model. These findings suggest that edaravone treatment would be effective in clinical patients recovering from cerebral infarction.

## 1. Introduction

The number of stroke patients has been increasing with the aging of the population. Among the causes of stroke, cerebral infarction is one of the most challenging diseases. Katrak et al. have reported that the prognoses of patients with cerebral infarctions were poor with less efficacy of rehabilitation compared to those with cerebral hemorrhages [1].

The free radical scavenger edaravone was approved in Japan in June 2001 for the treatment of cerebral infarctions [2]. Edaravone is currently widely used in clinics in Japan [3]. In recent years, combination therapy with tissue plasminogen activator and edaravone has begun, and functional improvements have been reported [4] with functional recovery effects in patients with lacunar infarctions [3, 5–7]. 

Although most animal experiments with infarction models have examined embolic models and their recanalization [8–14], few reports have examined models of thrombosis. In addition, there have been few studies of the time course of functional recovery. Thus, we investigated photochemically induced cerebral thrombosis in rats and observed the functional recovery and the infarcted area after the administration of the free radical scavenger.

## 2. Materials and Methods

All experimental procedures were conducted in accordance with the guidelines for animal experimentation of both Kagoshima University and the National Institutes of Health.

### 2.1. Animals

A total of 36 adult Wistar rats weighing 250–300 g were used in the behavior assessments and in the infarction area study after 7 days of infarction. In addition, 6 adult Wistar rats weighing 250–300 g were used in immunological assessments and the infarction area study after 24 hours of infarction.

### 2.2. Creating the Infarction

Cerebral ischemia was produced with intravascular thrombosis. This thrombosis was induced by an intravenous injection of rose bengal (20 mg/kg) and irradiation by green light (533 nm; MHF-G150LR, SCHOTT MORITEX Corporation, Saitama, Japan). The surgeries were performed while the animals were under general anesthesia with isoflurane (Foren; Abbott Japan Co., Ltd., Tokyo, Japan). In order to target the sensorimotor area of the cerebral cortex, green light with a 10 mm diameter was irradiated into the exposed skull 6 mm lateral to the midline and 4 mm posterior to bregma. Rose bengal was injected through the tail vein [15].

### 2.3. Edaravone Injection

After creating the infarction, the edaravone group was intraperitoneally injected with edaravone (3 mg/kg). The control group was injected with saline. 

### 2.4. Behavior Assessments

The recovery process of the hemiplegia was evaluated with the 7-step scale of Fenny [16]. The postoperative locomotion of the rats was evaluated with a beam-walking task on an elevated narrow beam (length: 1,220 mm; width: 25 mm). The performance was rated daily on a 7-point scale. The lowest score, 1, was given if the animal was unable to traverse the beam and could not place the affected hind limb onto the horizontal surface. A score of 2 was given if the animal was unable to traverse the beam but placed the affected hind limb on the horizontal surface of the beam and maintained balance. An animal received a score of 3 if it traversed the beam while dragging the affected hind limb. If an animal traversed the beam and placed the affected hind paw on the horizontal surface of the beam at least once, it was given a score of 4. A score of 5 was given if the animal used the affected limb in less than half of the steps along the beam. When an animal traversed the beam and used the affected limb to aid more than 50% of its steps along the beam, it received a score of 6. The highest score, 7, was given if the animal traversed the beam normally with fewer than 2 independent foot slips. Rats that were given a score of 1 the day after the operation were used in the subsequent analysis. We evaluated these rats daily from before the operation until 7 days after the operation.

### 2.5. Infarcted Brain Assessments

After perfusion fixation with 4% paraformaldehyde, the brains were removed, and the infarction area was measured the day after the infarction and 7 days after the infarction. The area of the infarction on the cerebral surface was imaged with the image analysis software (ImageJ, US National Institutes of Health, Bethesda, MD, USA). The statistical analyses were conducted with the Wilcoxon signed-rank test (*P* < 0.01). Hematoxylin-Eosin staining was performed on the fixed sections of the infarcted cortex on day 7 in order to confirm the infarction.

## 3. Results 

**Figure 1:**
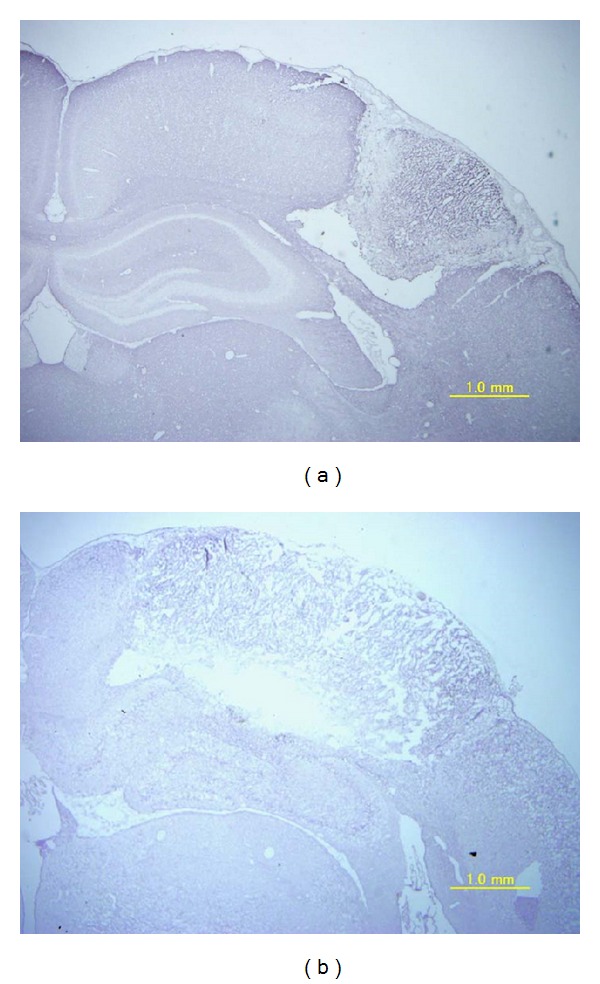
Photomicrograph of rat brain 7 days after photochemical infarction. Edaravone-administered (a) and control (b).

**Figure 2:**
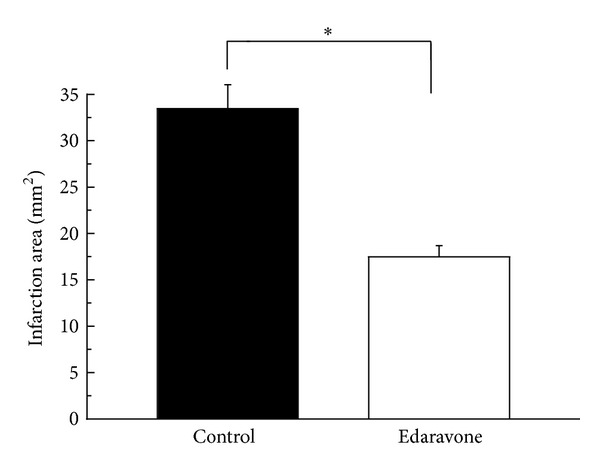
Areas of infarction 7 days after photochemical infarction (*P < 0.01).

**Figure 3:**
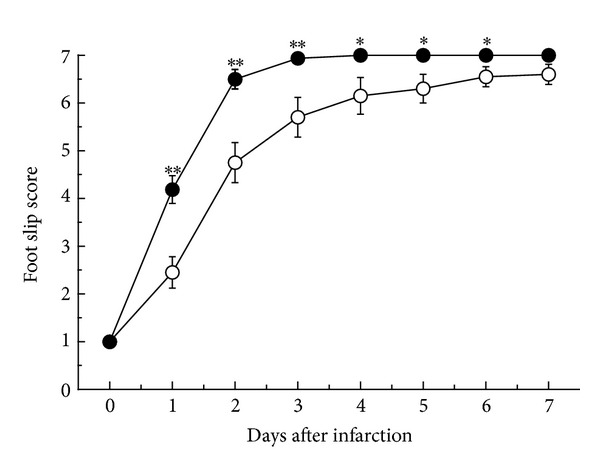
Functional recovery from photochemical infarction. The closed circles indicate the edaravone-treated group, and the open circles indicate the control group (*P < 0.05, **P < 0.01).

There were infarcted lesions on the irradiated sensorimotor cortex the day after the infarction and 7 days after the infarction. The infarction area after 7 days was smaller in the edaravone-treated group (Figure 1). The infarcted area 1 day after the infarction was 20.9 ± 4.90 (mean ± standard error of the mean (SE)) mm^2^ in the edaravone-treated group and 27.2 ± 8.02 mm^2^ in the control group. There was no significant difference between these groups. Seven days after the infarction, the area of infarction was 17.4 ± 1.39 mm^2^ in the edaravone group and 33.3 ± 2.66 mm^2^ in the control group. There was a significant difference between these groups (*P* < 0.0001, Figure 2). The recovery of paralysis in the edaravone-treated group was significantly earlier than in the untreated group. After 7 days, both groups were mostly recovered with scores of 7 (Figure 3). The scores for the beam walking test were 4.2 ± 0.29 on day 1 after infarction, 6.5 ± 0.2 on day 2, 6.93 ± 0.06 on day 3, and 7.00 ± 0.00 on day 4. However, the mean scores were 2.45 ± 0.32 on day 1, 4.75 ± 0.42 on day 2, 5.7 ± 0.42 on day 3, 6.15 ± 0.39 on day 4, 6.30 ± 0.30 on day 5, 6.55 ± 0.21 on day 6, and 6.6 ± 0.21 on day 7. 

## 4. Discussion

Free radicals gradually increase in the areas surrounding the ischemic area, including the penumbra, and they rapidly increase after reperfusion. OPC-14117 [17], NXY-097 [18], and other free radical scavengers have been previously examined. However, clinical applications of these have not been done, except with edaravone.

Abe et al. have conducted basic research on edaravone, and it was approved as a brain protection drug in 2001 in Japan [2, 19]. Edaravone has a protective effect on oxidative injuries in the brain by eliminating free radicals. It has been widely used in Japan in acute patients with cerebral thromboses and cerebral embolisms [3]. There have been reports that edaravone has protective effects on lacunar infarctions in clinical patients [5–7]. A recent study has reported that the administration of edaravone with cilostazol had more protective effects [20]. In order to increase blood flow and diminish free radicals, the coadministration of tissue plasminogen activator and edaravone or edaravone injections after the administration of tissue plasminogen activator have been shown to be effective in a clinical study [4, 21].

Most animal studies utilize rat embolic models or reperfusion models [8–12]. Besides rat models, rabbit models have also been reported. However, the rabbit models were embolic models [13, 14]. There have been few reports on the time course of the function of the extremities. Nishi et al. have evaluated rat activity with 5-grade scores, but a time-course study was not done [8]. Kawai et al. have evaluated hemiplegic rats with a 4-step qualitative analysis of abnormal postures when hanging by the tail. However, the time course was not evaluated [22]. Jin et al. have evaluated the survival rate, neuronal survival, and blood flow after the simultaneous administration of argatroban and edaravone in *Meriones unguiculatus*. This report was also silent about physical activities [23]. 

Recently, Lu et al. have reported the effects of edaravone on cognitive function that was evaluated by a maze test in a transient global ischemia model. There was early improvement in the edaravone-administered group, and the results were compatible with our results [24]. Zhang et al. have reported the effects of edaravone according to infarction size and improvements in physical activity [12]. These findings were also compatible with our results.

We studied the effects of edaravone in a rat model of photochemically induced infarction. There have been no reports concerning the effects of edaravone in a model of photochemically induced infarction. The distinctive feature of this model was that we were able to obtain similar infarction sizes and locations. It was presumed that the ischemic changes were similar to those of cerebral thrombosis and that we were able to compare it with other cerebral embolism models, because a thrombus was formed in the irradiated area of the cortex.

In our results, the infarcted areas were not significantly different between the edaravone-treated group and the control group the day after the infarction. However, the infarcted area in the edaravone group was about half of the size of that in the control group. The infarcted area was smaller in the edaravone-treated group than in the control group in the embolization model of middle cerebral artery occlusion [12]. These findings suggested that edaravone has inhibiting effects on infarction formation and reducing effects on the formation of infarction. 

In rat infarction models, the functional deficits are recovered within about 2 weeks after the infarction. Therefore, a time-course study is important in order to evaluate the effects of medication or rehabilitation. In this study, we detected an enhanced effect of edaravone on functional recovery in infarcted rats. 

Recently, edaravone has been investigated in global brain ischemia and transient ischemic attacks [24]. The combination therapy of edaravone with low temperatures has been reported [25]. There has been a report that edaravone was effective in neonatal cerebral ischemia [26] and epilepsy [27]. Edaravone has been studied in traumatic brain injury, spinal cord injury, neurodegenerative disease, and brain tumor [28–30]. Thus, edaravone is thought to have various effects on central nervous system disturbances. 

## 5. Conclusions

The free radical scavenger edaravone reduced the infarction area and promoted the functional recovery of hemiparesis from cerebral thrombosis in a rat model. These findings suggested that treatment with edaravone is effective for the recovery from cerebral infarctions in clinical patients.

## References

[1] Katrak PH, Black D, Peeva V (2009). Do stroke patients with intracerebral hemorrhage have a better functional outcome than patients with cerebral infarction?. *PM and R*.

[2] Watanabe T, Tanaka M, Watanabe K, Takamatsu Y, Tobe A (2004). Research and development of the free radical scavenger edaravone as a neuroprotectant. *Yakugaku Zasshi*.

[3] Lapchak PA (2010). A critical assessment of edaravone acute ischemic stroke efficacy trials: is edaravone an effective neuroprotective therapy?. *Expert Opinion on Pharmacotherapy*.

[4] Yamashita T, Kamiya T, Deguchi K (2009). Dissociation and protection of the neurovascular unit after thrombolysis and reperfusion in ischemic rat brain. *Journal of Cerebral Blood Flow and Metabolism*.

[5] Nakase T, Yoshioka S, Suzuki A (2011). Free radical scavenger, edaravone, reduces the lesion size of lacunar infarction in human brain ischemic stroke. *BMC Neurology*.

[6] Ohta Y, Takamatsu K, Fukushima T (2009). Efficacy of the free radical scavenger, edaravone, for motor palsy of acute lacunar infarction. *Internal Medicine*.

[7] Mishina M, Komaba Y, Kobayashi S (2005). Efficacy of edaravone, a free radical scavenger, for the treatment of acute lacunar infarction. *Neurologia Medico-Chirurgica*.

[8] Nishi H, Watanabe T, Sakurai H, Yuki S, Ishibashi A (1989). Effect of MCI-186 on brain edema in rats. *Stroke*.

[9] Liu N, Shang J, Tian F, Nishi H, Abe K (2011). In vivo optical imaging for evaluating the efficacy of edaravone after transient cerebral ischemia in mice. *Brain Research*.

[10] Watanabe T, Yuki S, Egawa M, Nishi H (1994). Protective effects of MCI-186 on cerebral ischemia: possible involvement of free radical scavenging and antioxidant actions. *Journal of Pharmacology and Experimental Therapeutics*.

[11] Wu T, Zeng L, Wu J, Fung K (2000). MCI-186: further histochemical and biochemical evidence of neuroprotection. *Life Sciences*.

[12] Zhang P, Li W, Li L (2012). Treatment with edaravone attenuates ischemic brain injury and inhibits neurogenesis in the subventricular zone of adult rats after focal cerebral ischemia and reperfusion injury. *Neuroscience*.

[13] Lapchak PA, Zivin JA (2009). The lipophilic multifunctional antioxidant edaravone (radicut) improves behavior following embolic strokes in rabbits: a combination therapy study with tissue plasminogen activator. *Experimental Neurology*.

[14] Lapchak PA (2010). Translational stroke research using a rabbit embolic stroke model: a correlative analysis hypothesis for novel therapy development. *Translational Stroke Research*.

[15] Horinouchi K, Ikeda S, Harada K (2007). Functional recovery and expression of GDNF seen in photochemically induced cerebral infarction. *International Journal of Neuroscience*.

[16] Feeney DM, Gonzalez A, Law WA (1982). Amphetamine, haloperidol, and experience interact to affect rate of recovery after motor cortex injury. *Science*.

[17] Abe K, Morita S, Kikuchi T, Itoyama Y (1997). Protective effect of a novel free radical scavenger, OPC-14117, on wobbler mouse motor neuron disease. *Journal of Neuroscience Research*.

[18] Bath PMW, Gray LJ, Bath AJG, Buchan A, Miyata T, Green AR (2009). Effects of NXY-059 in experimental stroke: an individual animal meta-analysis. *British Journal of Pharmacology*.

[19] Abe K, Yuki S, Kogure K (1988). Strong attenuation of ischemic and postischemic brain edema in rats by a novel free radical scavenger. *Stroke*.

[20] Yamamoto Y, Ohara T, Ishii R (2011). A combined treatment for acute larger lacunar-type infarction. *Journal of Stroke and Cerebrovascular Diseases*.

[21] Kamiya T, Abe K (2011). Future neuroprotective strategies in the post-thrombolysis era—neurovascular unit protection and vascular endothelial protection. *Clinical Neurology*.

[22] Kawai H, Nakai H, Suga M, Yuki S, Watanabe T, Saito K (1997). Effects of a novel free radical scavenger, MCI-186, on ischemic brain damage in the rat distal middle cerebral artery occlusion model. *Journal of Pharmacology and Experimental Therapeutics*.

[23] Jin Y, Mima T, Raicu V, Park KC, Shimizu K (2002). Combined argatroban and edaravone caused additive neuroprotection against 15 min of forebrain ischemia in gerbils. *Neuroscience Research*.

[24] Lu F, Nakamura T, Toyoshima T (2012). Edaravone, a free radical scavenger, attenuates behavioral deficits following transient forebrain ischemia by inhibiting oxidative damage in gerbils. *Neuroscience Letters*.

[25] Nito C, Kamiya T, Amemiya S, Katoh K, Katayama Y (2003). The neuroprotective effect of a free radical scavenger and mild hypothermia following transient focal ischemia in rats. *Acta Neurochirurgica, Supplementum*.

[26] Ikeda T, Xia YX, Kaneko M, Sameshima H, Ikenoue T (2002). Effect of the free radical scavenger, 3-methyl-1-phenyl-2-pyrazolin-5-one (MCI-186), on hypoxia-ischemia-induced brain injury in neonatal rats. *Neuroscience Letters*.

[27] Kamida T, Fujiki M, Ooba H, Anan M, Abe T, Kobayashi H (2009). Neuroprotective effects of edaravone, a free radical scavenger, on the rat hippocampus after pilocarpine-induced status epilepticus. *Seizure*.

[28] Itoh T, Satou T, Nishida S, Tsubaki M, Hashimoto S, Ito H (2009). The novel free radical scavenger, edaravone, increases neural stem cell number around the area of damage following rat traumatic brain injury. *Neurotoxicity Research*.

[29] Itoh T, Satou T, Nishida S (2010). Edaravone protects against apoptotic neuronal cell death and improves cerebral function after traumatic brain injury in rats. *Neurochemical Research*.

[30] Kikuchi K, Kawahara K, Uchikado H (2011). Potential of edaravone for neuroprotection in neurologic diseases that do not involve cerebral infarction. *Experimental and Therapeutic Medicine*.

